# White Matter Fiber Tracking Method with Adaptive Correction of Tracking Direction

**DOI:** 10.1155/2024/4102461

**Published:** 2024-02-05

**Authors:** Qian Zheng, Kefu Guo, Yinghui Meng, Jiaofen Nan, Lin Xu

**Affiliations:** ^1^Zhengzhou University of Light Industry, Zhengzhou, China; ^2^Chengdu University of Traditional Chinese Medicine, Chengdu, China

## Abstract

**Background:**

The deterministic fiber tracking method has the advantage of high computational efficiency and good repeatability, making it suitable for the noninvasive estimation of brain structural connectivity in clinical fields. To address the issue of the current classical deterministic method tending to deviate in the tracking direction in the region of crossing fiber region, in this paper, we propose an adaptive correction-based deterministic white matter fiber tracking method, named FTACTD.

**Methods:**

The proposed FTACTD method can accurately track white matter fibers by adaptively adjusting the deflection direction strategy based on the tensor matrix and the input fiber direction of adjacent voxels. The degree of correction direction changes adaptively according to the shape of the diffusion tensor, mimicking the actual tracking deflection angle and direction. Furthermore, both forward and reverse tracking techniques are employed to track the entire fiber. The effectiveness of the proposed method is validated and quantified using both simulated and real brain datasets. Various indicators such as invalid bundles (IB), valid bundles (VB), invalid connections (IC), no connections (NC), and valid connections (VC) are utilized to assess the performance of the proposed method on simulated data and real diffusion-weighted imaging (DWI) data.

**Results:**

The experimental results of the simulated data show that the FTACTD method tracks outperform existing methods, achieving the highest number of VB with a total of 13 bundles. Additionally, it identifies the least number of incorrect fiber bundles, with only 32 bundles identified as wrong. Compared to the FACT method, the FTACTD method reduces the number of NC by 36.38%. In terms of VC, the FTACTD method surpasses even the best performing SD_Stream method among deterministic methods by 1.64%. Extensive in vivo experiments demonstrate the superiority of the proposed method in terms of tracking more accurate and complete fiber paths, resulting in improved continuity.

**Conclusion:**

The FTACTD method proposed in this study indicates superior tracking results and provides a methodological basis for the investigating, diagnosis, and treatment of brain disorders associated with white matter fiber deficits and abnormalities.

## 1. Introduction

White matter fiber bundles are the complex structural composition of the human brain and the material basis for information exchange between functional brain regions [1]. The emergence of magnetic resonance diffusion tensor imaging technology provides significant advantages for studying the shape and distribution of white matter fiber bundles. Diffusion tensor imaging (DTI) and white matter fiber tract tracking technology have been widely used in the research of brain neurological diseases such as apoplexy [2–4], schizophrenia [5–7], and Parkinson's disease [8–10], which have made contributions to the physiological research and clinical research. In recent years, techniques have been continuously updated and iterated for studying white matter fiber tracts.

White matter fiber tracking technology has mainly gone through two development stages. The first stage is the use of traditional invasive research methods, such as anatomical staining, which require dissection and can only be limited to the study of human and animal cadavers, not suitable for in vivo research; the second stage is the noninvasive 3D reconstruction technology of living human brain white matter fiber anatomy, which is mainly divided into four categories: deterministic fiber tracking method, probabilistic fiber tracking method, global optimization method, and tracking method based on machine learning and deep learning. Classic and promising deterministic tracking methods have been proposed including fiber assignment by continuous tracking (FACT) [11], tensor deflection (TEND) [12], tensorline [13], and vector criterion tracking (VCT) [14]. Probabilistic fiber tracking methods model neighborhood information of the voxel in the image [15] to obtain the probability density function (PDF) or fiber orientation distribution (FOD) of fiber orientation. This type of method gives the probability of all possible fiber paths, takes the direction with the largest probability value as the fiber direction in the voxel, and then tracks and reconstructs the white matter fibers. This type of method uses Bayesian probability tracking [16] proposed by Friman et al. as the basic framework, second-order integration over fiber orientation distributions (iFOD2) [17], and later, some researchers proposed particle filtering tractography (PFT) [18] and unscented Kalman filter (UKF) [19] based on the filter. The global optimization tracking methods have been proposed by exploring the cost function, which represents the smoothness of the fiber or the goodness of fit of the measurement signal, and obtaining the fiber trajectory by optimizing the cost function, such as the Gibbs tracking method [20] and fiber tracking based on graph theory method [21]. With the development of machine learning and deep learning, Li et al. proposed a fiber tracking method based on machine learning [22], using random forests and decision trees to obtain the probability values of multiple sampling directions. Poulin et al. used a feedforward neural network and recurrent neural network to obtain the distribution function of white matter fiber direction and used a deterministic tracking method to describe the fiber. Probabilistic fiber tracking can improve the comprehensiveness of fiber distribution and describe more complex fiber structures, which will cause more pseudo fibers. Screening out pseudo fibers is also a challenge so far, so specific nerve fiber paths cannot be visualized. Moreover, probabilistic fiber tracking is sensitive to reaching the white matter boundary, which leads to termination prematurely. In addition, a large amount of random sampling is required, which is computationally intensive and inefficient. The tracking speed currently cannot meet clinical requirements. The advantage of the global optimization method is that it produces fewer false fibers, but it has a large amount of calculation and the tracking results are rough. Tracking methods based on machine learning and deep learning need to use the tracking results of other methods as training samples, which takes a long time, and the tracking results are prone to overfitting. Compared with the other three types of methods, the deterministic fiber tracking method is intuitive and easy to understand with less calculation and execution time, which meets the needs of clinical applications.

Although a multitude of deterministic fiber tracking methods has been proposed over the last few decades, there are some potential shortcomings of these methods. For example, the FACT method considers the trajectory of a fiber bundle in the human brain as a spatial three-dimensional curve. If the direction of each pixel on the curve is considered as the tangential direction of that pixel, then this vector corresponds to the main feature vector of that point, and the main direction of the feature vector is the direction of travel of the voxels. This method is relatively simple and easy to understand, and it can fit the fiber travel path well in the region with high fractional anisotropy. However, in the region of low fractional anisotropy, the tracking results will produce large deviations. Moreover, the tracking results are susceptible to noise, which makes the tracked fibers rough. The TEND method first starts from the center point of a voxel in the predefined region of interest (ROI) and advances along the direction of the main feature vector of the voxel. When reaching the boundary of the next voxel, the fiber advancing direction at this time is calculated by the function of the diffusion tensor (DT) of the voxel. The method uses the entire diffusion tensor to deflect the estimated fiber trajectory, which improves tracking accuracy. However, TEND method deflects all voxels, which is good for the areas with small fractional anisotropy (FA), but the tracking effect in areas with high FA values is not as good as the FACT method. The tensorline method is the weighted average of the earliest streamline tracking (STT) and the TEND method. By adjusting the weight parameters to determine the next fiber tracing direction, the method can effectively utilize both methods. However, the weight parameters need to be set by yourself, which is difficult to determine. The VCT method estimates the direction of the next step of fiber tracking by calculating the principal eigenvectors of the surrounding adjacent voxels and the distance from the center point of each voxel to the point to be determined. If the angle between them is greater than the set threshold, the direction is excluded, and the distance between its center point and the point to be determined is set to 0, thereby excluding the influence of the main eigenvector of this point on the entire fiber direction. However, the method considers that only eight neighboring voxels in 3D space can affect the fiber tracking direction of this fixed point, without considering all neighboring voxels that may affect the direction of this point. It is easy to cause the fiber tracking direction to deviate from the correct direction. To deal with the above problem, we propose a white matter fiber tracking method with adaptive correction of tracking direction, named FTACTD. In this method, the fiber direction of the target voxel is no longer corrected uniformly but is corrected differently for different voxels.

## 2. Materials and Methods

**Figure 1:**
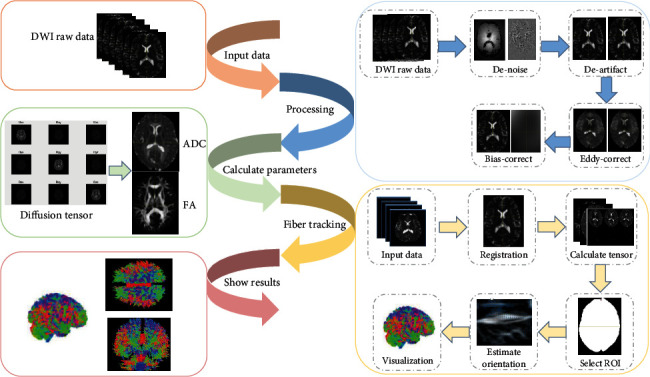
Fiber tracking technology roadmap.

The steps of the white matter fiber tracking method for adaptively correcting the tracking direction mainly include data preprocessing, computing the DT, eigenvector, and FA. White matter nerve fiber tracking is realized by adaptively correcting the fiber tracking direction, and the results of fiber tracts are visualized and analyzed. The roadmap of the proposed fiber tracking technology is given in Figure 1.

### 2.1. Data Collection

The clinical dataset can qualitatively evaluate and demonstrate the effect of fiber tracking, but the real human brain dataset lacks ground truth, and the tracking results cannot be compared quantitatively. The simulated dataset can quantitatively evaluate the results of fiber tracking and calculate quantitative indicators; thus, in this paper, we used a simulated dataset and a real human brain dataset to evaluate the tracking results qualitatively and quantitatively. The simulated dataset used in this paper was released at the ISMRM (International Society for Magnetic Resonance in Medicine) 2015 Challenge (https://tractometer.org/ismrm2015/dwi_data/). This simulated data was generated by Fiberfox software based on the fiber bundles segmented by the Human Connectome Project (HCP) dataset in the United States, and it is currently the only simulated dataset that can perform quantitative index calculations. The voxel size is 2 mm × 2 mm × 2 mm, the whole-brain size is 90 × 108 × 90, and the gradient includes 1 diffusion sensitivity coefficient *b*‐value = 0 s/mm^2^ and 32 diffusion-weighted images in the gradient direction of *b*‐value = 1000 s/mm^2^. The real human brain dataset derives from the MRI data of 50 adult subjects, and each subject was scanned with the same parameters as described below. DWI data were acquired using a single-shot echo-planar imaging sequence with the following parameters: repetition time/echo time = 8900/95 ms, matrix size = 128 × 128 (FOV 256 mm × 256 mm), 1.8 mm isotropic resolution, 40 DWI directions, and *b*‐value = 1000 s/mm^2^, with 7 *b* = 0 images; voxel size is 2.1875 mm × 2.1875 mm × 2.2 mm.

### 2.2. Preprocessing

The existence of large amounts of random noise, artifacts, and geometric distortion caused by magnetic susceptibility in DWI images can affect the accuracy of fiber tracking and lead to fiber tracking interruption. A series of preprocessing is needed to be performed to obtain more accurate DWI data before fiber tracking, so a more accurate binary mask image can be obtained, which will benefit for improving the tracking accuracy and obtaining a more continuous fiber bundle path. The experiment uses the same preprocessing steps for the simulated dataset and the real human brain dataset, and the preprocessing is implemented on the MRtrix platform (https://www.mrtrix.org/).

The preprocessing steps for DWI images are as follows.

*Step 1 (DWI denoising)*. There is noise and distortion in the original DWI data, and the influence of noise can be reduced by using the denoise command, which estimates MRI noise level and denoises based on random matrix theory.

*Step 2 (Gibbs artifact removal)*. This artifact, also known as truncation artifact, is related to spatial resolution. It is well known that an image consists of very small pixels and contains an infinite number of spatial frequencies, but the system only collects image signals at a limited number of frequencies leading to Gibbs artifacts, which can be removed from DWI images using local subvoxel displacement methods.

*Step 3 (DWI distortion correction using dwifslpreproc)*. Correct the geometric distortion caused by the magnetic susceptibility present in the diffusion image, as well as any distortion caused by eddy currents and the subject's main body motion, and the step depends on the FSL command.

*Step 4 (B1 field inhomogeneity correction for a DWI volume series)*. This step is meant to improve brain mask estimation. However, if no strong bias fields are present in the data, running this script might deteriorate brain mask estimation and result in inferior brain mask estimation.

### 2.3. The Diffusion Tensor and Fractional Anisotropy Calculation

#### 2.3.1. Diffusion Tensor (DT)

Basser and Jones [23] proposed a method to calculate DT using NMR spin echo. DT is not represented by scalar values, but by introducing a 3 × 3 second-order tensor matrix to describe the ellipsoidal diffusion model of water molecules in three-dimensional space. There are six independent components, with the diagonal elements representing the displacement along the orthogonal axis and the off-diagonal elements representing the correlation with the orthogonal displacement. *S* is the signal intensity measured when the diffusion gradient direction *g* is applied, which is the obtained original diffusion-weighted images. *S*_0_ is the signal intensity measured without applying any gradient direction pulse, which is the diffusion-weighted image without the diffusion gradient direction. *b* is the value of the diffusion gradient, which is a known constant and determined by the experimental conditions. In the formula, *D* is the apparent diffusion coefficient, also known as ADC, which is an independent component of DT and represents the diffusion ability of water molecules [24]. The calculation of the DT is given as

(1) $$\begin{array}{c} S_{i}=S_{0}\times e^{-{bD}_{i}}. \end{array}$$

Take logarithmic transformation on both sides of the above formula, and the following formula can be obtained:

(2) $$\begin{array}{c} {\text{ADC}}_{i}=-\frac{1}{b}\ln\frac{S_{i}}{S_{0}}. \end{array}$$

This means that without considering the noise, the 6 independent components of DT can be uniquely determined by doing 7 independent experiments: *S*_0_ was measured once without applying any diffusion-sensitive magnetic field; the other 6 times were to add the gradient of the diffusion-sensitive magnetic field in 6 noncollinear directions *e*_*k*_ = (*x*_*k*_, *y*_*k*_, *z*_*k*_)^*T*^(*k* = 1, 2, 3, 4, 5, 6) and use the same *b* value to measure and obtain 6 different diffusion-weighted images, respectively; that is, a six-variable linear algebraic equation system is obtained, and DT can be obtained by solving the equation.

#### 2.3.2. Fractional Anisotropy (FA)

One of the most common parameter indexes used to describe the diffusion characteristics of different tissue structures of the brain is FA [25]. FA is the ratio of the anisotropy to the entire DT, reflecting the anisotropy of the diffusion of water molecules, and the range is 0-1, which can be formulated as follows:

(3) $$\begin{array}{c} \text{FA}=\sqrt{\frac{3}{2}}\sqrt{\frac{{\left(\lambda_{1}-\bar{\lambda }\right)}^{2}+{\left(\lambda_{2}-\bar{\lambda }\right)}^{2}+{\left(\lambda_{3}-\bar{\lambda }\right)}^{2}}{\lambda_{1}^{2}+\lambda_{2}^{2}+\lambda_{3}^{2}}}. \end{array}$$

#### 2.3.3. Geometric Measures of Diffusion

Westin et al. [26] used three characteristic indices to describe the linear, planar, and spherical diffusion models relatively completely. The calculation formula is shown in

(4) $$\begin{array}{c} \text{Cl}=\frac{\lambda_{1}-\lambda_{2}}{\lambda_{1}},\\ \text{Cp}=\frac{\lambda_{2}-\lambda_{3}}{\lambda_{1}},\\ \text{Cs}=\frac{\lambda_{3}}{\lambda_{1}}, \end{array}$$

where Cl + Cp + Cs = 1; Cl represents the degree of linear anisotropy, Cp represents the degree of planar anisotropy, and Cs represents the degree of isotropy.

### 2.4. Proposed FTACTD Method

Most deterministic fiber tracking methods only consider the DT local information and directly take the direction of the voxel main eigenvector as the direction of fiber tracking. This method is more accurate only when the diffusion tensor model of the voxel is an ellipsoid. If the diffusion tensor model of voxels is in the shape of a disk or sphere, this method will result in significant errors. Some methods will correct the current fiber tracking direction concerning the tracking direction of the previous step and the current DT, but the method deflects the tracking direction of all voxels. In voxels with small FA, better results can be achieved, while in voxels with large FA, excessive deflection can easily occur, leading to error accumulation. Therefore, considering the advantages and disadvantages of the above-mentioned methods, we propose a white matter fiber tracking method with adaptive correction of tracking direction. The method adaptively selects the tracking direction based on the diffusion characteristics of the target voxel. During the fiber tracking process, instead of simply progressing along a fixed direction, the method dynamically chooses the direction based on the current voxel's diffusion characteristics. The specific implementation is as follows: for the target voxel, the Cl and Cp are first computed. These coefficients reflect the characteristics of the diffusion tensor in different directions. When the voxel's eigenvalues *λ*_1_ ≫ *λ*_2_ ≈ *λ*_3_, resulting in a high FA, and Cp tends to be 0, the latter part of the formula approaches 0. The tracking direction is determined by the former part of the formula. At this point, it is the tracking that progresses along the direction of the principal eigenvector. On the other hand, when *λ*_1_ ≈ *λ*_2_ ≫ *λ*_3_, Cl tends to be 0, and the fiber tracking direction is influenced by the latter part of the formula. In this case, there is a high probability that it is a fiber intersection area, which will cause uncertainty in the fiber tracking direction. Therefore, based on the current voxel's diffusion coefficients and the current diffusion tensor model, a correction is applied to the previous tracking direction to adapt to the current voxel's diffusion shape. This ensures the continuity of fiber tracking. Additionally, due to the consideration of the previous fiber tracking direction, the next tracking direction is computed based on the previous step. Therefore, when tracking passes through the target voxel again, due to the difference in the direction of the previous step, crossing fibers in other directions can be tracked. The method proposed in this paper departs from the conventional approach of having all voxels progress solely along the direction of the principal eigenvector, addressing the limitation of tracking only one fiber in a voxel. Thus, the algorithm introduced in this paper offers greater flexibility in dealing with the complexity of fiber structures, especially in regions of fiber crossings, thereby enhancing the accuracy and continuity of fiber tracking. The specific process is to select the voxel in the ROI (select the entire brain as a region of interest) that satisfies the FA greater than the specified threshold as the seed point and track in the direction of the main eigenvector of this point. When reaching the boundary of the next voxel, the tracking direction is modified adaptively through the values of Cl and Cp. When the value of Cl is larger, the tracking direction adopts the direction of the main eigenvector. When the value of Cp is larger, the main feature vector of the voxel is deflected according to the previous tracking direction and the tensor matrix of the current voxel, which is used as the next tracking direction. When the values of Cl and Cp are between 0 and 1, the fiber tracking direction is the sum of the weights of the direction of the main feature vector and the direction of the previous tracking direction deflected by the diffusion tensor of the current voxel. In addition, it is necessary to trace along the negative direction from the seed point for getting a complete fiber bundle. Finally, the tracking results along the positive and negative directions are merged into a complete fiber path. The FTACTD method can calculate the next fiber tracking direction *V*_out_ as follows:

(5) $$\begin{array}{c} V_{\text{out}}=\text{Cl}\times e_{1}+\text{Cp}\times D\times V_{\text{in}}. \end{array}$$

That is,

(6) $$\begin{array}{c} V_{\text{out}}=\frac{\lambda_{1}-\lambda_{2}}{\lambda_{1}}\times V_{1}+\frac{\lambda_{2}-\lambda_{3}}{\lambda_{1}}\times D\times V_{\text{in}}, \end{array}$$

where *V*_out_ is the fiber tracking direction, *V*_1_ is the main eigenvector direction of the current voxel, *D* is the diffusion tensor of the current voxel, and *V*_in_ is the tracking direction of the fiber of the previous adjacent voxel.

**Figure 2:**
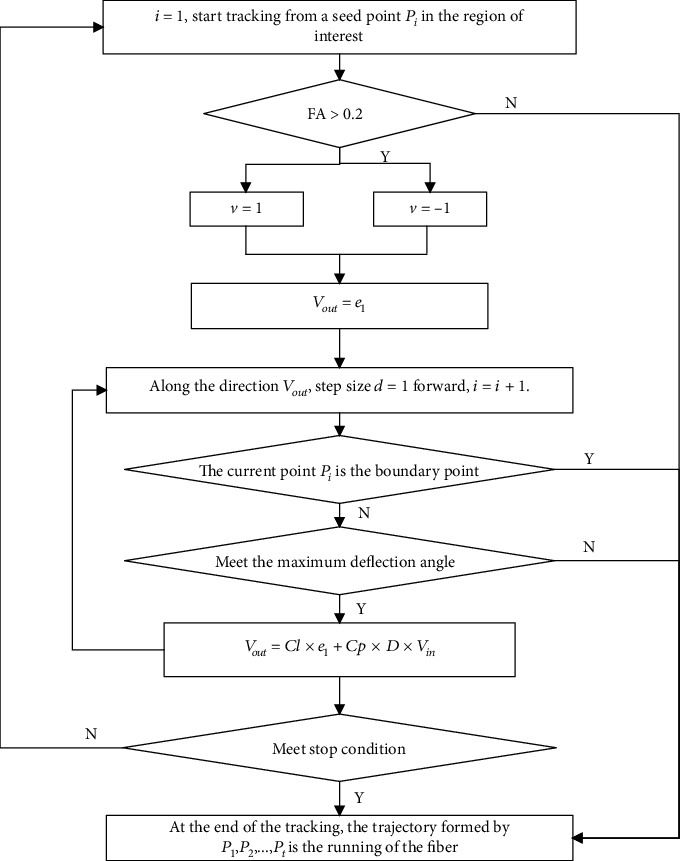
Flowchart of the proposed FTACTD method.

The specific flowchart of the FTACTD method is given in Figure 2.

## 3. Results

The performance of the FTACTD is compared against FACT, TEND, streamline tractography based on spherical deconvolution (SD_Stream) [27], iFOD2, and anatomically constrained tractography second-order integration over fiber orientation distributions (ACT_iFOD2) [28] to provide relevant comparisons using simulated data ISMRM 2015 and 50 cases of real human brain dataset. FACT, TEND, and SD_Stream are popular deterministic fiber tracking methods, and iFOD2 and ACT_iFOD2 are probabilistic fiber tracking methods. In addition, FACT and TEND use the DTI model, and SD_Stream and the two probabilistic fiber tracking methods use the constrained spherical deconvolution (CSD) model. The selection of the optimal tracking parameters in FTACTD is given in Appendix.

### 3.1. Simulation

**Figure 3:**
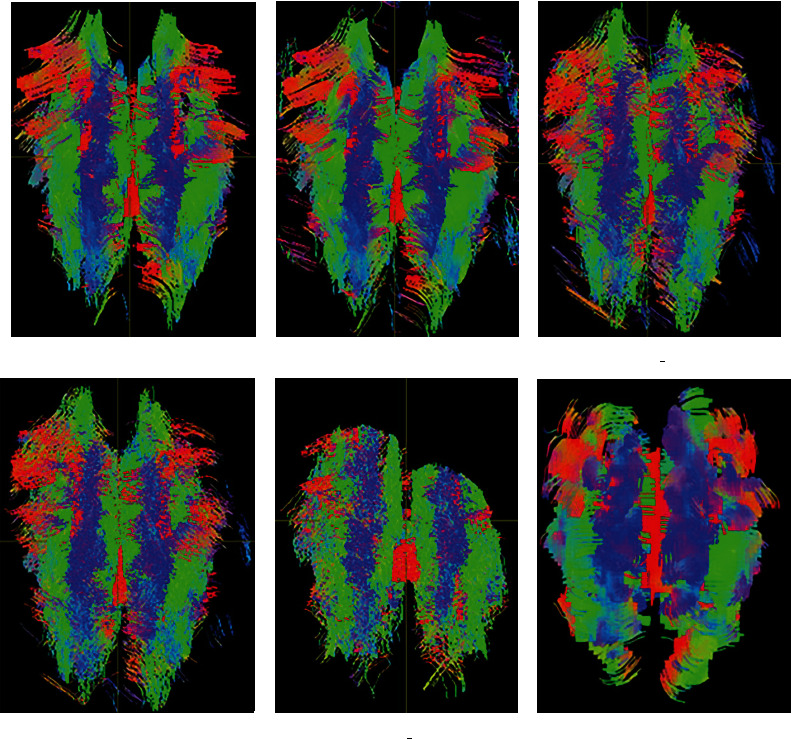
Comparison of tracking effects of whole-brain regions of the simulated dataset.

Figure 3 provides the visual experimental results of fiber distribution in the whole brain for the evaluation of fiber tracking methods under the simulated dataset. By comparing with the anatomical atlas, the distribution of nerve fiber bundles tracked by all methods is consistent with the distribution of anatomical nerve fibers. The smoothness of the fiber bundles tracked by SD_Stream and iFOD2 methods is the worst, whereas the FTACTD method proposed in this paper is the best compared with the other fiber tracking methods. From the perspective of fiber distribution, TEND and SD_Stream methods produce a large number of messy and wrong fibers distributed on both sides of the left and right brain, while the tracking results of the FTACTD method have no messy and wrong fiber distribution. Comparing the results of the iFOD2 and ACT_iFOD2 methods, it can be seen that the ACT step can reduce the number of fibers greatly and also reduce a lot of effective fibers, indicating that the combination of the ACT method can greatly affect the tracking effect of the method.

The Tractometer [29] (https://tractometer.org/), an independent evaluation tool of the ISMRM 2015 Challenge, was used to evaluate and compare the performance of fiber tracking methods quantitatively. The quantitative measures provided by Tractometer [30] are invalid bundles (IB), valid bundles (VB), invalid connections (IC), no connections (NC), and valid connections (VC).

Table 1 presents the discrete values of the VB, VC, IC, NC, and IB on the simulated dataset by using different fiber tracking methods under investigation. It can be seen that the tracking result accuracy of the improved method proposed in this paper is higher than that of the FACT method and the TEND method. Specifically, the FTACTD method can track more VB compared with the FACT method and the TEND method. In terms of IC, the minimum IC value obtained by the tracking result of the FACT method is 23.26%. In terms of VC, the value obtained by the tracking result of the FACT method is also the smallest, and the proposed improved FTACTD method has the largest value obtained by the tracking result. Compared with the FACT method, the VC of the FTACTD method has increased by 3.9%, proving that the improved method tracks the least amount of incorrect directions. In terms of NC, the minimum value obtained by the FTACTD method is 36.76%, while the maximum value obtained by the FACT method is 73.14%, which may be because the tracking fails to reach the termination area (such as prematurely touching the boundary and terminates). In terms of IB, the results obtained by the FTACTD method and ACT_iFOD2 method are far less than that of the FACT method and TEND method. The IB value obtained by the ACT_iFOD2 method is the smallest, because this method adds the ACT method based on the iFOD2 method, and this method will delete some wrong fibers that do not conform to anatomy. Existing deterministic fiber tracking methods such as FACT and TEND only consider the information of the voxel itself. Probabilistic fiber tracking methods such as iFOD2 and ACT_iFOD2 only consider the influence of neighboring voxels, resulting in large errors in the final tracking results. FTACTD is a deterministic fiber tracking method that not only considers the diffusion structure of the voxel itself but also selects the next tracking direction according to the fiber history and can achieve better tracking results.

### 3.2. In Vivo Imaging

**Figure 4:**
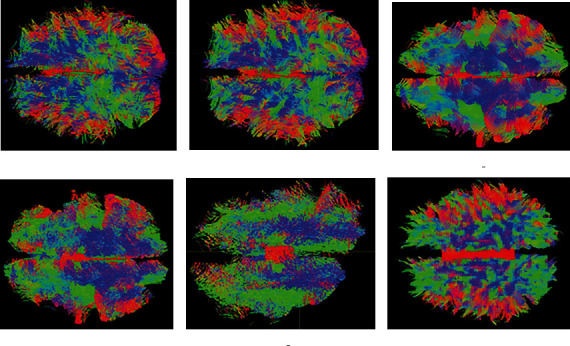
Comparison of tracking effects of whole-brain regions of real human brain dataset.

This section is devoted to verifying the consistency of the proposed FTACTD approach on in vivo dataset, using the real human brain dataset of 50 subjects to compare the FTACTD method with the other five methods and present the statistical parameters of the tracking results of 50 subjects in the form of mean ± standard deviation. However, due to space constraints, only one case of the dataset is selected for visualization of fiber tracking results. The fiber tracking results of the proposed FTACTD and the other five different tracking methods in the whole human brain are presented in Figure 4. This figure shows that the fiber tracking results achieved through the above six methods can clearly display the physiological structure characteristics of fiber bundles. Moreover, compared with the anatomical map, the tracked whole-brain fiber bundles are basically consistent with the anatomical nerve fibers. However, compared with the other five methods, the fibers tracked by the FTACTD method are smoother and have fewer messy fibers. This shows that for some branched nerve fibers with small angles, the FTACTD method can make appropriate choices according to the overall fiber running trend.

**Figure 5:**
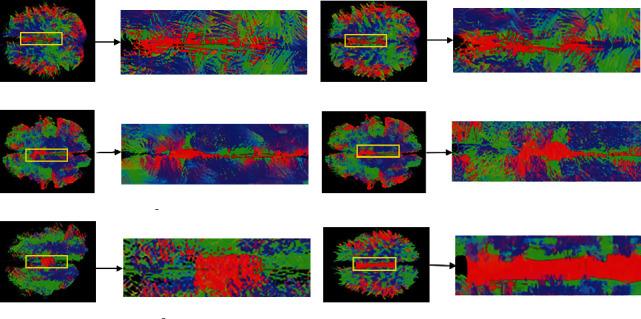
Partial enlarged view of junction area.

**Figure 6:**
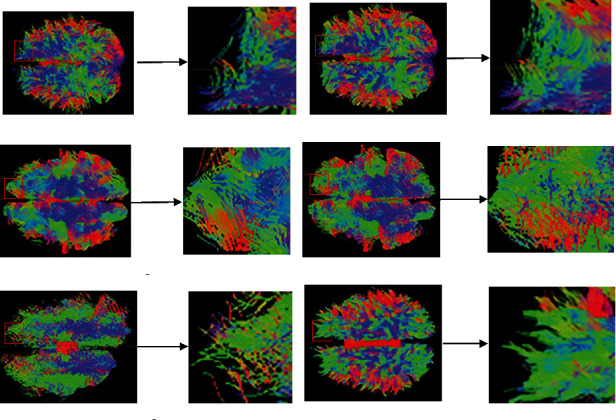
Partial enlarged view of frontal pole.

To provide a better visual inspection, Figures 5 and 6 show the enlarged details of using a real human brain dataset to track the entire brain region. The association fiber mainly connects the corresponding functional regions of the left and right hemispheres, which are transverse nerve fiber bundles connecting the left and right cerebral hemispheres, located in the figure's middle region of the left and right hemispheres. From Figure 5, we can see that the FTACTD method tracks the association fiber more completely and continuously. Although the FACT and TEND methods also tracked the commissural fibers in the middle, the fibers at both ends were messy, some truncated fibers existed, and the tracking was incomplete. The SD_Stream and iFOD2 methods track based on the CSD model, which enables the method to obtain more comprehensive fiber structure information, but correspondingly produces a lot of pseudo fibers. Therefore, the association fiber is covered by other traveling fibers due to the method tracking many small fibers and wrong fibers. The tracking results of the ACT_iFOD2 method are based on the ACT method after the basic screening of the iFOD2 method results. We can see that only part of the association fiber is tracked by the method, and many correct commissural fibers have been deleted. The green fiber bundles represent the fiber bundles that run forward and backward. Combined with the physiological structure characteristics of the fiber bundles, the left and right ends of the picture should be dominated by the green fiber bundles that run forward and backward. It can be seen from Figure 6 that the FTACTD method conforms to this physiological feature, followed by the TEND method. Although TEND also traced out the fiber bundles with green directions at both ends, they were not continuous. The tracking results of the FACT method in the regions at both ends are mixed with a variety of colors, and a large number of other fiber bundles are distributed. It can be seen that the tracking effect of the method is worse than that of the other two methods. The results tracked by iFOD2 and SD_Stream are messy at both ends. From the ACT_iFOD2 method, it can be seen that in the left part of the tracking result, many green fiber bundles are running back and forth, but many correct fiber bundles are missing in the right part.

There is no ground truth in the real human brain dataset to support the quantitative calculation of the Tractometer, so this section only counts the results of whole-brain nerve fiber tracking of 50 subjects and presents them in the form of mean ± standard deviation.

The statistical results are presented in Table 2. It can be seen that the results of the deterministic tracking method and the probabilistic tracking method are quite different. It can be seen from the deterministic tracking method that the method proposed in this paper can track more fibers than the FACT and TEND methods, and the average length of the tracked fibers is longer. The statistical results of the SD_Stream method are quite different from those of the other three deterministic methods. This is because the SD_Stream is based on the CSD model, while the other three deterministic methods are based on the DTI model. From the results of the probabilistic fiber tracking method, the iFOD2 tracked the most fiber bundles. This is consistent with the principle that the probabilistic tracking method can obtain a more comprehensive fiber distribution, and the number tracked by the SD_Stream method is second. This is because both the SD_Stream and iFOD2 methods track based on the same model. Although the ACT_iFOD2 method is also a probabilistic tracking method, the method adds an ACT step and deletes wrong fibers from the results of iFOD2, so the average fiber number tracked by this method is 15847 fewer than that of the iFOD2 method. However, the probabilistic tracking method takes a long time, and the average tracking time of the ACT_iFOD2 method reaches 153.6 seconds. From the overall dataset, the average fiber length tracked by the FTACTD method proposed in this paper is the longest among the six methods, reaching 120.92 mm. In addition, the running time of the FTACTD method is significantly reduced compared with the probabilistic tracking method, which is in line with the acceptable range of clinical applications.

## 4. Discussion

Three popular deterministic fiber tracking methods and two probabilistic fiber tracking methods are selected for experimental comparison and evaluation with the FTACTD method proposed in this paper. Experiments on the simulated dataset and the collected real human brain dataset demonstrate that the FTACTD method outperforms current state-of-the-art methods in white matter fiber tracking.

In the deterministic fiber tracking method, the fiber tracking direction of each step in the tracking process is uniquely determined. Among them, the TEND method and the FACT method are fiber tracking based on the DTI model. The DTI model can only produce more accurate direction information in areas with a high degree of anisotropy. Therefore, this type of method can achieve better tracking results for single direction tracking. But it is difficult to track complex intersections and bifurcation regions. The difference between the FACT method and the TEND method lies in the direction of fiber tracking in the next step. The FACT method takes the direction of the main eigenvector of the seed point as the direction of the fiber bundle, advances until it reaches the boundary of the next voxel, and then selects the direction of the main eigenvector of the next voxel to track and continue to move forward until the termination condition of certain thresholds is met. This method one-sidedly considers that each voxel contains only one fiber running direction, which is the direction of the main eigenvector. This method is only suitable for areas with strong anisotropy. The TEND method starts from the center point of a voxel in the artificially selected ROI and advances toward the main eigenvector of the voxel. When the boundary of the next voxel is reached, the forward direction of the fiber is calculated by the DT of the next voxel. In regions with less anisotropy, the tracking direction of the voxel no longer follows the direction of the main eigenvector. However, in the region with a high degree of anisotropy, the deflection at this time will cause the fiber tracking direction to be wrong. Although SD_Stream is also a deterministic tracking method, it is essentially different from the microstructure models of the above three types of methods. It is based on the HARDI model [31] for fiber tracking. The HARDI model can describe the direction information of complex areas, so the method can be used in complicated areas to trace out the correct fiber orientation (such as crossing areas).

Probabilistic fiber tracking selects the fiber tracking direction through the direction probability, and different fiber directions may be selected according to the probability, to describe the comprehensive information of the fiber. Although the tracking results are relatively comprehensive, a large number of false fibers are produced, which is consistent with the experimental results and very messy. The iFOD2 method advances along a fixed-length step and is tangent to the current tracking direction at the current point. It considers the FOD of the candidate points on each candidate path, and the curve with the highest combined probability corresponds to the most likely path (probability calculation on each path of iFOD2 is a product of each infinitesimal step probability). The ACT_iFOD2 method is a combination of the ACT method and the iFOD2 method. The ACT method does not affect the fiber tracking process but directly affects the fiber tracking results. By combining T_1_-weighted (T_1_w) and DWI to obtain a more accurate mask and incorporating anatomical prior knowledge, the tracking results of the iFOD2 method are screened to remove unreasonable fibers. It can be seen that many fibers in the tracking results of the iFOD2 method have been removed, but by comparing the results of deterministic fiber tracking, it can be seen that the ACT method has also removed a large number of effective fibers, which is the biggest limitation of the ACT method. Moreover, the method is closely related to the gradient direction of the acquired DWI, and the dataset requirements are relatively high. These objective reasons will directly affect the tracking effect of the method.

The method proposed in this paper uses Mrtrix3-related commands in the preprocessing stage to perform head-movement correction, distortion correction, noise reduction, artifact, and bias correction on the dataset, which improves the tracking accuracy of the method. In biology and neuroscience, fiber tracts exist as both crossing fibers and single-directional fibers. Based on this characteristic, a novel fiber tracking strategy is proposed. From DTI fiber tractography, it is known that when the FA are high, fiber tracking proceeds along the direction of the principal eigenvector. When the FA approaches 0, water molecules undergo isotropic diffusion, such as in the gray matter (GM) and cerebrospinal fluid (CSF) regions, where it is assumed that no fiber bundles exist. Additionally, there are cases of disk-shaped diffusion, where fiber crossings are prevalent. Therefore, ignoring the complexity of fiber structures and tracking solely along the direction of the principal eigenvector or correcting the tracking direction for all voxels to adapt to the diffusion model can lead to cumulative errors. The FTACTD method takes into account the complexity of fiber structures and adaptively adjusts the tracking direction based on the diffusion tensor model of voxels. In DTI-based fiber tractography, the diffusion direction of water molecules is commonly employed as the tracking direction for fibers. Therefore, when *λ*_1_ ≫ *λ*_2_ ≈ *λ*_3_, the diffusion tensor model of a voxel takes on an ellipsoidal shape, indicating that water molecules diffuse along the direction of the principal eigenvector, spreading toward both ends of the ellipsoid. In such cases, the direction of the principal eigenvector is chosen as the tracking direction for the fibers. When *λ*_1_ ≈ *λ*_2_ ≫ *λ*_3_, the diffusion tensor model of a voxel appears disk-shaped, and water molecules may diffuse in the directions corresponding to *λ*_1_ and *λ*_2_, potentially resulting in fiber crossings and bifurcations. In such situations, the problem of fiber direction selection needs to be considered. Simply using the direction of the principal eigenvector may lead to errors in tracking direction. Therefore, when the diffusion tensor shape of a voxel resembles a disk-shaped, this paper introduces consideration for the previous tracking direction. By utilizing the shape of the diffusion tensor and diffusion coefficients, a certain degree of deflection is applied to the previous tracking direction. This helps align the tracked fibers more with the smoothness characteristics. Moreover, as the tracking path passes through the target voxel again, the change in the previous tracking direction leads to a corresponding change in the next tracking direction, enabling the tracking of crossing fibers. Therefore, this dynamic adjustment of the tracking path and adaptive approach to the complex fiber structure in tissue can achieve more accurate fiber tracking.

In the simulated dataset, from the distribution of whole-brain fibers, the method proposed in this paper has the best and smoothest tracking effect. Judging from the calculation results of quantitative indicators, the FTACTD method tracks the largest number of VB, the VC is also the highest among all methods, and the NC achieves the minimum value of 36.76%. This is because the FTACTD method can well adapt to the tracking of curved fibers and can effectively avoid premature fiber termination caused by premature contact with the boundary. Moreover, the FTACTD method no longer only tracks along the direction of the main eigenvector but makes different corrections to the fiber tracking direction according to the actual situation of each voxel. Therefore, the method can track fewer wrong directions, making VC higher and NC lowest.

In the real human brain dataset, from the perspective of the overall distribution of fibers, the fibers tracked by the tracking method FTACTD proposed in this paper are longer and more complete. The smoothness is also the best among the six methods, and the symmetry of the nerve fibers of the left and right hemispheres is also better. The above advantages can be seen from local regions such as association fiber connecting the left and right hemispheres and green fiber bundles located at both ends of the brain. From the perspective of association fiber, we can see that the FTACTD method proposed in this paper can trace the most complete and continuous commissural fibers than the other five methods, and the tracking results of the other five methods are messier or have truncated fibers. From the green fiber bundles located in the anterior and posterior courses at the two ends of the brain, the FTACTD method clearly shows a large number of green fiber bundles distributed at the two ends of the resultant map, which is consistent with the physiological anatomy features. The ACT_iFOD2 method distributes many green fiber bundles on the left end of the resulting image, but the right-end fiber is seriously missing. The other four methods have unsatisfactory tracking results in this respect, especially the tracking results of the FACT method distribute other traveling fiber bundles in the two end regions, and the effect is the worst. This is because the FTACTD method makes corrections for each voxel tracking direction differently, which significantly reduces the error caused by taking the main eigenvector direction as the fiber running direction. The tracking direction in the case of planar diffusion is improved, and the fiber tracking in strong and weak anisotropy areas can be well completed. Moreover, the FTACTD method comprehensively considers the historical trend of the fiber and the local information of the current tensor to determine the next fiber tracking direction. Therefore, the FTACTD method can achieve better tracking results than the other five methods.

The limitation of the proposed FTACTD method is that the used fiber microstructure reconstruction models are center-symmetric, but the actual fiber distribution is usually asymmetric. Thus, fiber tracking based on asymmetric models is required to be developed in the future.

## 5. Conclusion

We presented FTACTD, a deterministic white matter fiber tracking method with adaptive correction of tracking direction based on the tensor matrix and the input fiber direction of adjacent voxels. In the quantitative and qualitative evaluations on the simulated dataset and real human brain dataset, the proposed method outperformed the state-of-art classical methods. Therefore, the method proposed in this paper can lay a methodological foundation for the research, diagnosis, and treatment of brain diseases caused by the loss and abnormality of white matter fibers.

## Figures and Tables

**Figure 7:**
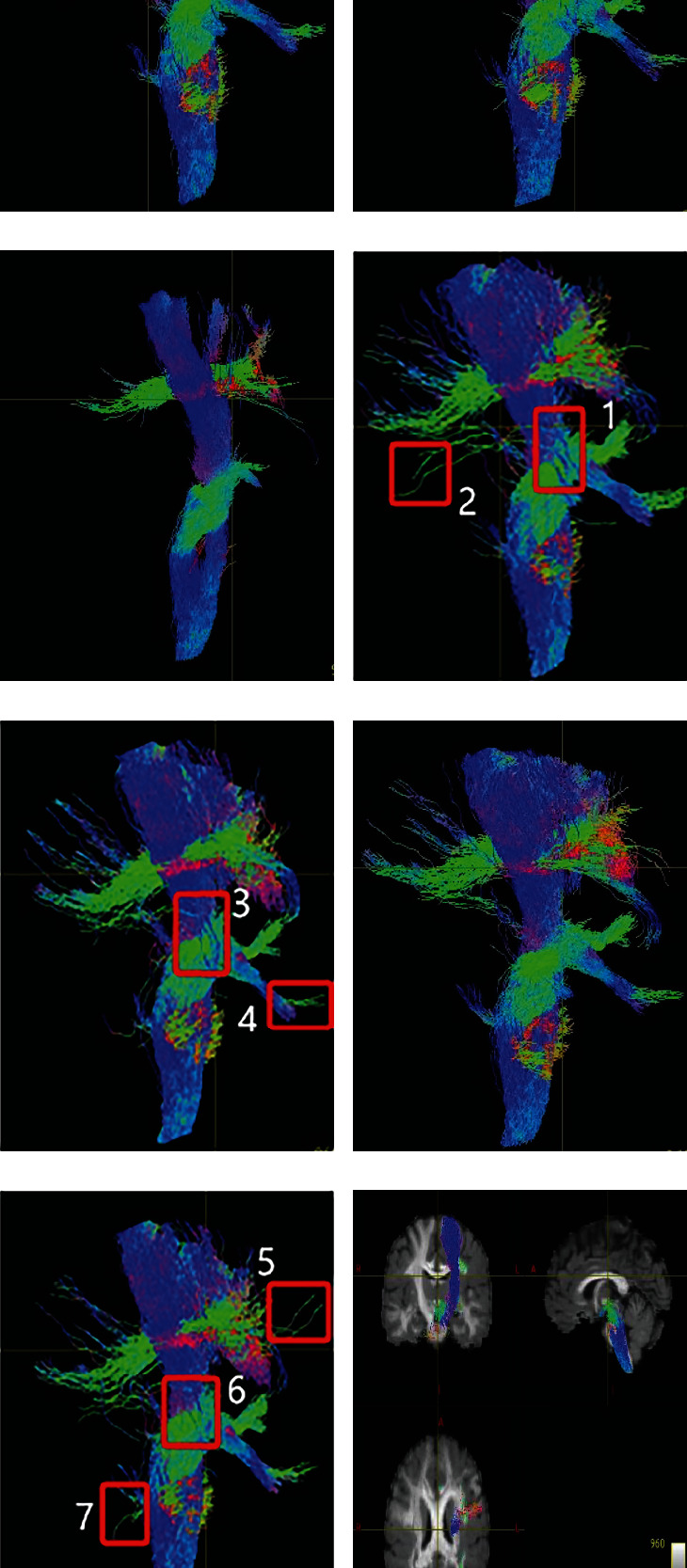
Comparison of tracking effects with different tracking parameters.

**Table 1:** VB, VC, IC, NC, and IB comparisons of different methods on the simulated dataset.

Methods	VB (bundle)	VC (%)	IC (%)	NC (%)	IB (bundle)
FACT	12	3.6	23.26	73.14	40
TEND	11	4.79	35.28	59.93	39
SD_Stream	11	5.86	41.72	54.42	42
iFOD2	11	5.72	47.98	46.3	38
ACT_iFOD2	10	5.36	42.86	51.78	22
FTACTD	13	7.5	55.74	36.76	32

Note: the VB in ground truth is 25 beams.

**Table 2:** Statistical comparison of the whole-brain tracking fiber parameters of the six methods ($\bar{x}\pm S$).

Methods	FB_num	FL_max	FL_min	FL_mean	Time
FACT	20544 ± 1029	469.69 ± 45.17	51.4 ± 16.11	115.6 ± 10.63	30.9 ± 3.9
TEND	10595 ± 766	510.89 ± 16.88	21.36 ± 8.21	119.3 ± 12.6	34.3 ± 4.1
SD_Stream	29562 ± 2704	102.74 ± 6.63	20.18 ± 5.39	22.49 ± 3.18	33.1 ± 2.3
iFOD2	41924 ± 3119	124.10 ± 6.10	20.39 ± 6.70	24.97 ± 4.27	96.9 ± 4.7
ACT_iFOD2	30286 ± 1663	215.34 ± 9.81	20.13 ± 10.7	48.04 ± 8.07	153.6 ± 8.6
FTACTD	22021 ± 2240	597.98 ± 20.45	52.54 ± 8.41	120.92 ± 10.8	39.92 ± 2.0

**Table 3:** Comparison of different fiber parameter settings and statistical results.

Group	FA	Angle	Step	Min	Max	Mean	Counts
a	0.2	45°	1 mm	10.81 mm	143.03 mm	41.15 mm	5115
b	0.1	45°	1 mm	10.85 mm	137.4 mm	40.23 mm	6286
c	0.3	45°	1 mm	10.83 mm	88.24 mm	28.33 mm	4986
d	0.2	45°	0.5 mm	10.37 mm	140.46 mm	40.47 mm	5163
e	0.2	45°	2 mm	11.77 mm	133.45 mm	40.53 mm	5000
f	0.2	50°	1 mm	10.83 mm	138.43 mm	40.83 mm	5112
g	0.2	60°	1 mm	10.74 mm	88.24 mm	42.28 mm	5107

## Data Availability

We gratefully acknowledge the public data for validating and quantifying the proposed method and have shared the download link to the data in the paper.

## Appendix

### Parameter Sensitivity Analysis of DTI Fiber Tracking

To verify the impact of tracking parameters on tracking performance, we selected values within the commonly used parameter range in the field of fiber optic tracking and conducted experiments using the FTACTD method. Selected parameters include 0.1, 0.2, and 0.3 for the FA; 0.5 mm, 1 mm, and 2 mm for the step size; and 45°, 50°, and 60° for the deflection angles. The experimental setting parameters and corresponding experimental statistical results are listed in Table 3.

Due to the excessive number of fibers in the whole-brain region, the visualization results do not show significant discrepancy. Therefore, we selected an area commonly used in experiments, with the left corticospinal tract (CST) as the main area for visualization (the location in the brain is shown as Figure 7(h)). The results of fiber tracking using different tracking parameters are shown in Figure 7.

Upon comparison of the three groups (a, b, and c), it is evident that when the FA is set to 0.3, the fiber count in group c significantly decreases compared to the other groups. This phenomenon is attributed to the fact that in DTI-based fiber tractography, tracking stops when FA falls below a certain threshold. Therefore, setting FA to 0.3 results in premature termination, causing the tracked fibers to be shorter. Moreover, when tracking from seed points, fibers may be missed if the voxel's FA is less than 0.3.  Table 1 shows that setting the FA to 0.3 results in only 4986 fibers, with a maximum fiber length of 88.24 mm. Group c has significantly shorter fibers, with an average length of approximately 28 mm, compared to groups a and b. Setting FA to 0.1 leads to an increase in tracking times, and the number of fibers increases by almost 1100 compared to other groups. This is particularly noticeable in the area above the fiber distribution in group b, where a large number of short and messy fibers are obviously added. In summary, excessively small FA results in an increase in the number of erroneous fibers, and the fibers exhibit a disorganized distribution in the boundary regions. Therefore, better tracking results can be setting FA to 0.2.

When comparing groups (a, d, and e), it is observed that group d has a higher number of unbundled wrong fibers at the position marked by red box 1. This may be due to a step size that is too small, making the tracking more sensitive to noise or local variations, leading to the presence of some erroneous fibers. Additionally, fibers marked at red box 2 are only present in group d, which could be attributed to overly fine tracking that captures erroneous fibers. It should be noted that a smaller step size can significantly increase the tracking time and demand more computational resources. Furthermore, it is important to balance the choice of step size to optimize resource utilization while maintaining accuracy. Table 3 shows that a small step size does not alter the fiber tracking results. When the step size is set to 2 mm, computational speed improves but tracking accuracy decreases, resulting in the loss of some fine or rapidly changing fibers. For example, in region 4 (marked by the red box in group e), the tracked fiber distribution is not as comprehensive as in groups a and d. In addition, there are some unbundled error fibers in the area marked by red box 3. In our experiments, we found that the method tracks the shortest fibers at 11.77 mm when the step size is 2 mm, possibly due to the risk of losing fine fibers caused by the larger step size. By appropriately choosing the step size, we can mitigate the impact of noise in the data and reduce the generation of erroneous fibers. Therefore, we ultimately set the tracking step size to 1 mm.

By comparing groups (a, f, and g), it can be observed that an appropriate of deflection curvature setting can eliminate some unreasonable fibers. For instance, in group g, marked in red box 6, a deflection curvature of 60° results in unbundled and unreasonable fibers, whereas in group a, setting the deflection curvature to 45° appears to remove these scattered fibers. Furthermore, at the locations marked in red boxes 5 and 7, group g exhibits overly curved fibers. Anatomically, fiber bundles are generally smooth, and any abrupt changes in the trajectory of fiber tracking are considered to be caused by noise or artifacts. When the deflection curvature is set to 50°, which is close to 45°, the visual tracking results are similar to those obtained at 45°. However, statistical parameters indicate that setting the deflection curvature to 45° yields better results in terms of the shortest fiber length, longest fiber length, and average fiber length. In our experiments, we select 45° as the deflection curvature termination condition for fiber tracking.

In relation to the selection of the region of interest (ROI), since the comparative methods and the FTACTD method in the experiment utilize the entire brain as the ROI, with the whole-brain white matter mask serving as seed points for executing whole-brain fiber tractography, there is no need to consider the discrepancy in fiber tracking results due to ROI. Supplementary explanations have already been included in the paper regarding the chosen ROI region in the experiment.
